# TRPV4 Plays a Role in Matrix Stiffness-Induced Macrophage Polarization

**DOI:** 10.3389/fimmu.2020.570195

**Published:** 2020-12-14

**Authors:** Bidisha Dutta, Rishov Goswami, Shaik O. Rahaman

**Affiliations:** Department of Nutrition and Food Science, University of Maryland, College Park, MD, United States

**Keywords:** macrophage, TRPV4 agonist, matrix stiffness, polarization, inflammation

## Abstract

Phenotypic polarization of macrophages is deemed essential in innate immunity and various pathophysiological conditions. We have now determined key aspects of the molecular mechanism by which mechanical cues regulate macrophage polarization. We show that Transient Receptor Potential Vanilloid 4 (TRPV4), a mechanosensitive ion channel, mediates substrate stiffness-induced macrophage polarization. Using atomic force microscopy, we showed that genetic ablation of TRPV4 function abrogated fibrosis-induced matrix stiffness generation in skin tissues. We have determined that stiffer skin tissue promotes the M1 macrophage subtype in a TRPV4-dependent manner; soft tissue does not. These findings were further validated by our *in vitro* results which showed that stiff matrix (50 kPa) alone increased expression of macrophage M1 markers in a TRPV4-dependent manner, and this response was further augmented by the addition of soluble factors; neither of which occurred with soft matrix (1 kPa). A direct requirement for TRPV4 in M1 macrophage polarization spectrum in response to increased stiffness was evident from results of gain-of-function assays, where reintroduction of TRPV4 significantly upregulated the expression of M1 markers in TRPV4 KO macrophages. Together, these data provide new insights regarding the role of TRPV4 in matrix stiffness-induced macrophage polarization spectrum that may be explored in tissue engineering and regenerative medicine and targeted therapeutics.

## Introduction

The essential role of biomechanical forces in regulating cell fate and behavior is increasingly becoming apparent (1–12). Such mechanotransduction is also implicated in all stages of tissue development of an organism starting from egg activation and organogenesis to maintenance of tissue homeostasis (13–16). Among known biomechanical cues, substrate stiffness (or rigidity) has sparked the interest of researchers because of its growing sense of involvement in various cellular processes such as cell adhesion, spreading, differentiation, migration, and proliferation (1–12). For example, mesenchymal stem cells have been reported to transduce changes in matrix stiffness into phenotypic changes and lineage specification (17). Other reports show that endothelial cells exhibit markedly enhanced proliferation rates when cultured on rigid hydrogels compared to soft hydrogels (18). Previous reports by our laboratory and others showed that numerous macrophage responses including differentiation, motility, phagocytosis, and proliferation are sensitive to matrix stiffness (5, 6, 19–26).

Tissue stiffness is thought to be governed in part by the stiffness of the underlying extracellular matrix (ECM). During inflammatory conditions, the ECM undergoes prolific remodeling that changes its stiffness and nano-characteristics. Such changes alter and compromise the function of tissues in pathological conditions like tumor development, fibrosis, atherosclerosis, and foreign body response (27–29). Idiopathic pulmonary fibrosis, a chronic fibro-inflammatory condition, is characterized by increased lung stiffness (3, 27, 30). Findings from the bleomycin-induced mouse model of fibrosis have shown that increased stiffness induces increased proliferation of lung fibroblasts, which is accompanied by considerable reduction in activity of autocrine inhibitors of fibrogenesis such as COX2 (27). The enhanced ECM stiffening in turn amplifies the fibrotic process in a positive feedback loop (31). In atherosclerosis, substratum stiffening is thought to increase membrane permeability and subsequent cholesterol entry, contributing to plaque development (29). These results suggest that excessive changes in the ECM can also directly regulate the phenotype and functions of immune cells like macrophages leading to deregulation of inflammatory processes (32–35).

Macrophages are found in almost all tissues, where they display a diverse array of activation states depending on spatio-temporal variations in the tissue microenvironment and associated stimuli (36). Classically activated pro-inflammatory M1 macrophages are typically induced by IFN(interferon)-*γ*, TNFα, and lipopolysaccharides, and express numerous markers including TNFα, IL(interleukin)-1α, IL-1β, IL-6, IL-12, iNOS, and low levels of IL-10 in addition to reactive oxygen and nitrogen species (37–39). Alternatively activated alternative-inflammatory (pro-healing) M2 macrophages are polarized by IL4 and IL13, and express numerous markers including IL10, CD36, Arg1, Mrc1, Ym1, and low levels of IL12 that aid in suppression of inflammation, increased wound healing, tissue remodeling, and angiogenesis (39–41). Owing to their diverse distribution, macrophages are subjected to wide variations in tissue stiffness ranging from 17 Pa in fat to 310 mPa in tendons (42). Over the years, several studies have aimed to understand the role of substrate stiffness on phenotypic changes in macrophages. Among the findings, when bone marrow-derived macrophages are grown on higher stiffness (840 kPa) poly (ethylene glycol)-RGD gels, they increased expression of proinflammatory cytokines such as TNFα, IL1β, and IL6, whereas cells grown on 130 kPa or 240 kPa gels did not (5). Other studies reported an apparently conflicting result, in which an alternative-inflammatory phenotype was induced in macrophages by growth on high stiffness 3D collagen-based matrices (43).

Over the past decade efforts have been made to elucidate the molecular mechanisms by which these biomechanical cues are integrated in the macrophage to modulate its polarization spectrum. One focus of this work has been on the role of mechanosensitive ion channels, in particular, the TRP (Transient Receptor Potential) cation channels in many macrophage functions including macrophage polarization spectrum (44–47). Previous reports by our laboratory and others showed that TRP channel of the vanilloid superfamily 4 (TRPV4) is a ubiquitously expressed, polymodal ion channel that can be activated by a diverse array of biochemical and biophysical stimuli including heat, osmotic stimuli, phorbol derivatives, growth factors, and matrix stiffness (7, 9, 20, 21, 25, 26, 30, 48–50). TRPV4 is linked to numerous pathophysiological functions including osmolarity sensing, shear-stress sensing in arteries, neurological responses, inflammation, tissue fibrosis, and osteogenesis (9, 20, 21, 26, 30, 48–58). We have recently identified a role of TRPV4 in various macrophage functions such as foam cell formation and foreign body giant cell formation in pathological conditions (20, 21, 26, 58). We found that TRPV4-mediated mechanotransduction integrated stiffness and lipopolysaccharide-induced signals to mediate enhanced uptake of oxidized low-density lipoprotein (26). Thus, it seemed possible that TRPV4 played a role in matrix stiffness-induced M1 and M2 polarization spectrum of macrophages. We have addressed this possibility here using an *in vivo* bleomycin-induced mouse skin fibrosis model. We found that skin stiffening was dependent on TRPV4 as evidenced by atomic force microscopy analysis. We found that augmented stiffness primed macrophages in the skin predominantly toward a M1 phenotype, and this priming was abrogated by genetic ablation of TRPV4. *In vitro* M1 polarization spectrum by a pathophysiologically-relevant matrix stiffness alone or in combination with soluble factors was also dependent on TRPV4. The loss of expression of matrix stiffness-induced M1 markers in TRPV4 KO macrophages was restored by TRPV4 reintroduction, suggesting an essential role of TRPV4 in macrophage polarization spectrum.

## Materials and Methods

### Reagents and Antibodies

Antibodies for iNOS (Catalog# 2982, RRID: AB-1078202) and Arginase-1 (Catalog# 93668, RRID: AB-2800207) were purchased from Cell Signaling Technology, Danvers, MA, USA. For immunohistochemistry, antibody for iNOS (Catalog# MA5-17139; RRID: AB 2538610) was purchased from Thermo Fisher Scientific (Waltham, MA, USA). ImmPact DAB solution, Peroxidase (Catalog# SK-4105) and Vector Red Substrate, Alkaline Phosphatase (Catalog# SK-5100) were purchased from Vector laboratories (Burlingame, CA, USA). All the primers used for quantitative reverse transcription polymerase chain reaction (qRT-PCR), One-Step Quantitative Reverse Transcription PCR kit, CD68 antibodies (Catalog# MCA1957, RRID: AB-322219), and F4/80 antibodies (Catalog# MCA497R, RRID: AB-323279) were obtained from BioRad (Hercules, CA, USA). RNeasy micro kit was purchased from Qiagen (Hilden, Germany). CP-qp-Cont-BSG colloidal probes and glass bottom 35 mm petri-dishes were obtained from sQube (Bickenbach, Germany) and WPI (Sarasota, FL, USA), respectively. Alexa-Fluor conjugated rat and rabbit secondary antibodies were purchased from Millipore-Sigma (Burlington, MA, USA). Mouse IL4, IL-13, and IFNγ were obtained from R&D Systems (Burlington, MA, USA), and *E*. *coli* lipopolysaccharide (LPS) (tlr1-3pelps) was purchased from InvivoGen (San Diego, CA, USA). Bleomycin was purchased from Hospira Inc. (Lake Forest, IL, USA). Mouse Ad-TRPV4-WT and Ad-Vec control constructs containing Adenovirus particles were generated by Vector Biolabs (Malvern, PA, USA). All cell culture related reagents were purchased from Thermo Fisher Scientific (Waltham, MA, USA).

### Animals, Thioglycolate-Induced Macrophages, and Adenovirus Infection

TRPV4 knockout (KO) mice on a C57BL/6 background originally generated by Dr. Makoto Suzuki (Jichi Medical University, Tochigi, Japan) were obtained from Dr. David X. Zhang (Medical College of Wisconsin, Milwaukee, WI, USA). Congenic WT mice were purchased from Charles River Laboratories (Wilmington, MA, USA). All animal experiments were performed following University of Maryland review committee-approved protocols in accordance with guidelines of the Institutional Animal Care and Use Committee (IACUC). We had used a male to female ratio of 3:2. Skin fibrosis was induced in 6 months old WT and TRPV4 KO mice by bleomycin injection, as previously described (57). Since aging is a known risk factor for fibrosis and its progression, aged mice (6 months old) were studied for bleomycin-induced skin fibrosis. We have used 8–10 weeks old mice for implantation-induced stiffness model by subcutaneous implantation of soft or stiff collagen-coated polyacrylamide hydrogels, as we previously published (20). Mice were housed and bred in a temperature and humidity controlled, pathogen-free environment with food and water provided *ad libitum*. Thioglycolate-induced macrophages were isolated 96 h after intraperitoneal injection of 2 ml of thioglycolate solution (90000-294; BD Bioscience), as described previously (59). Macrophages were cultured in 10% fetal bovine serum-supplemented DMEM (Catalog# 11995065, Gibco). The adenovirus vector, Ad(RGD)-CMV-mTRPV4 (Ad-TRPV4-WT), used in our study, was generated by Vector Biolabs (Malvern, PA, USA). We used human adenovirus Type 5 (dE1/E3) with RGD-fiber modification that contains mouse TRPV4 transgene under the control of cytomegalovirus major immediate early (CMV) promoter as viral backbone. The virus has a deletion in two genes E1 and E3 to render them replication incompetent. For our study, we used a titer of 10^8^ PFU/ml. For TRPV4 overexpression, macrophages were infected with Ad-TRPV4-WT or Ad-Vec (control) constructs for 48 h. Freshly prepared macrophages have been first seeded on collagen-coated 50 kPa substrate and then infected by Ad-TRPV4-WT, or Ad-Vec (control) constructs for 48 h.

### Bleomycin and Implantation-Induced Skin Tissue Stiffness Models

Bleomycin-induced skin tissue stiffness was achieved by daily injection of bleomycin or saline subcutaneously for 28 days as described previously (57). Briefly, 100 μl bleomycin (10 mg/kg) was injected subcutaneously into the shaved back of mice (n = 5 mice/group) every alternate day for 28 days. Skin tissue surrounding the injected region was collected for analysis. Implantation-induced stiffness model was generated by subcutaneous implantation of soft (1 kPa) or stiff (50 kPa) collagen-coated polyacrylamide hydrogels (Matrigen, Brea, CA, USA) on both flanks of the mice. The implants and the adjacent tissue were surgically removed from all the mice 28 days post-implantation, as described previously (20).

### Reverse Transcription Quantitative Polymerase Chain Reaction (qRT-PCR)

Total RNA was extracted from thioglycolate-induced macrophages using the RNeasy Micro kit (Qiagen) following instructions of the manufacturer. Using the One-Step Quantitative Reverse Transcription PCR kit and following the standard protocol provided by the manufacturer, we performed qRT-PCR using the following primers: *Il1β* (qMmuCEP0054181), *Mcp-1* (qMmuCED0003785), *iNOS* (qMmuCID0023087), *Il6* (qMmuCED0045760), *Tnfα* (qMmuCED0004141), *Arg1* (qMmuCID0022400), *Il10* (qMmuCED0044967), *Cd36* (qMmuCID0014852), *Mrc1* (qMmuCED0045846), *Ym1* (qMmuCID0022826), and *gapdh* (qMmuCEP0039581). Expression of a gene was determined as the amount of the gene relative to mRNA for GAPDH using the comparative C_T_ method described in the Bio-Rad qRT-PCR system user bulletin.

### Immunofluorescence and Immunohistochemistry (IHC) Analysis

Frozen skin tissue sections (7 µm) were incubated with anti-CD68 (1:100), anti-iNOS (1:100), anti-Arginase 1 (1:100), and anti-F4/80 (1:100) at 4°C overnight to identify protein expression and localization. Anti-Rat IgG conjugated with Alexa-Flour 594 (1:200) and anti-rabbit IgG conjugated with Alexa-Flour 488 (1:200) were used as secondary antibodies for immunofluorescence. Nuclei were stained with DAPI. Digital immunofluorescence intensity was quantified using ImageJ software (NIH), and results are presented as Integrated (Int) Density (the product of Area and Mean Gray Value) (20). DAPI staining was used to identify nuclei. For IHC analysis, 7 μm sections were incubated with primary Abs (anti-CD68, anti-iNOS, or Arg-1) for 1 h at room temperature after applying peroxidase blocking solution (3% hydrogen peroxide) for 5 min. Slides were washed in PBS with Tween (PBST; 0.025% Triton-X 100; 3× for 5 min) before adding the “polymer” HRP- or alkaline phosphatase (AP)-conjugated secondary Abs (Vector Laboratories). Sections were incubated with secondary Ab for 60 min on a shaker, followed by three PBST washes, 5 min each. Sections were then incubated with chromogenic substrates. We quantified the number of both iNOS and Arg-1-positive macrophages in 40× images to determine the ratio of iNOS-positive macrophages to Arg-1-positive macrophages.

### Atomic Force Microscopy (AFM)

For AFM analysis of stiffness, skin tissue sections (30 µm) were immobilized on glass slides in PBS. All stiffness measurements of skin tissue were performed using a JPK Nanowizard 4 AFM (Bruker Nano GmbH, Berlin, Germany) combined with an inverted optical microscope (Nikon Eclipse TE200, Melville, NY, USA). A CP-qp-Cont-BSG cantilever with a nominal resonance frequency of 30 kHz in air, spring constant of 0.1 N/m, and gold coating on the detector side with a length of 125 µm were used. A borosilicate glass sphere with a nominal diameter of 10 µm was attached to the cantilever tip. Using thermal noise fluctuations, the spring constant of the cantilever was calibrated before every measurement. A total of 70 individual contact mode force spectroscopic curves were generated across 5 areas in bleomycin- or saline-treated tissues independently with an approach speed of 2 µm/s and a maximum setpoint of 0.7 nN per condition. The Young’s modulus was calculated using JPK Data Processing software by fitting individual contact mode force spectroscopic curves into the Hertz model.

### Statistical Analysis

All statistical analysis was performed using GraphPad v7.0. Data are presented as mean ± standard deviation. Student’s *t*-test or one-way ANOVA followed by Bonferroni test was used to compare two independent variables; ns, not significant, *p < 0.05, **p < 0.01, and ***p < 0.001.

## Results

### TRPV4 Plays a Role in Skin Tissue Stiffening Under Fibrotic Conditions

**Figure 1:**
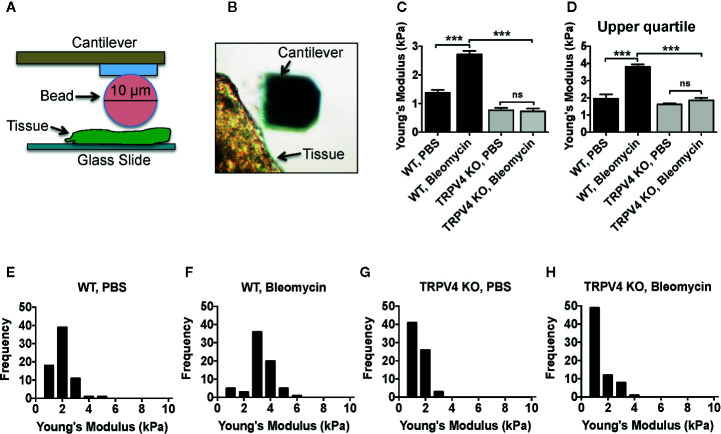
Fibrosis-induced stiffening of skin tissue is reliant on TRPV4. **(A)** Schematic diagram of atomic force microscopy (AFM) set up to determination the stiffness (Young’s modulus) of skin tissue. Deflection of a laser beam by deformation of the cantilever attached to a 10 µm borosilicated glass bead was recorded by a detector. A force curve generated by this process was fitted to the Hertz model to achieve Young’s modulus value. **(B)** Representative optical microscopic image shows a tissue section and cantilever. **(C)** Quantification of Young’s modulus in tissues under different conditions, and **(D)** upper quartile data points derived from the histograms shown in **(E–H)**. **(E–H)** Frequency distribution of Young’s modulus measurement of skin tissues collected from bleomycin or saline-treated WT and TRPV4 KO mice. n = 5 tissue samples per group and 70 force maps per sample. One-way ANOVA followed by Bonferroni’s test; *ns*, not significant; ***p < 0.001.

Subcutaneous injection of bleomycin, a widely used profibrotic drug, was used to induce tissue stiffening in both WT and TRPV4 KO mice. Previously we reported that TRPV4 plays a role in bleomycin-induced skin and pulmonary fibrosis at histological and biochemical levels (30, 57). Here, we directly measured bleomycin-induced skin tissue stiffness using atomic force microscopy (AFM). The AFM cantilever with a spherical tip of 10 µm was used to indent the skin tissue (**Figure 1A**). Cantilever deflection was detected by a position-sensitive detector (**Figure 1B**). Tissue sections were chosen carefully to be sufficiently close to the fibrotic area while also displaying heterogeneity of the tissue. We found WT tissues treated with bleomycin developed significant increases in stiffness (~2.71 kPa) compared to saline treated tissues (~1.38 kPa) (**Figures 1C–H**). However, deficiency of TRPV4 significantly decreased stiffness of both bleomycin and saline-treated tissues (0.73 and 0.77 kPa) (**Figures 1C–H**). Since stiffer areas are likely to show greater effects of mechanotransduction, upper quartile stiffness values were also measured, and were found to follow a similar trend (**Figure 1D**). Further analysis showed that WT tissue stiffness was distributed between 3 and 4 kPa in bleomycin-treated tissues, whereas stiffness of saline-treated tissues was ≤2 kPa (**Figures 1E–H**). Interestingly, genetic ablation of TRPV4 completely abolished the heterogeneity of tissue stiffness, skewing values to 1 kPa or lower in both treated and untreated conditions (**Figures 1E–H**). Taken together, these direct stiffness measurements indicate that TRPV4 is indispensable for skin stiffening under fibrotic conditions.

### TRPV4 Deficiency Differentially Regulates M1 and M2 Marker Expression *In Vivo* in Response to Varied Matrix Stiffness

**Figure 2:**
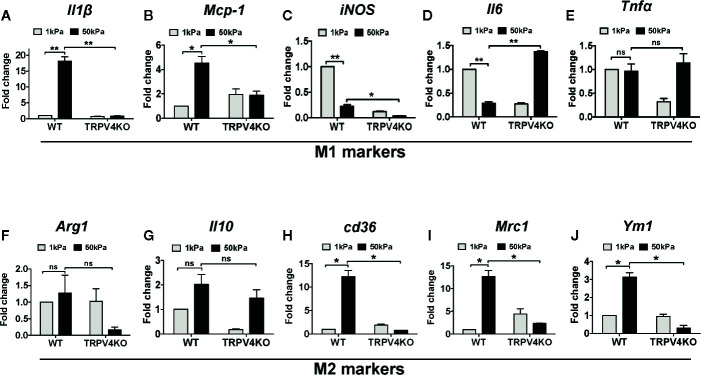
Deficiency of TRPV4 function differentially regulates M1 and M2 marker expression *in vivo* in a matrix stiffness-dependent manner. Total mRNA was harvested from 1 and 50 kPa collagen-coated polyacrylamide hydrogel implanted skin tissues of WT and TRPV4 KO mice. Expression (qRT-PCR analysis) of *Il1β* **(A)**, *Mcp-1* **(B)**, *iNOS* **(C)**, *Il6* **(D)**, *TNFα* **(E)**, *Arg1* **(F)**, *Il10* **(G)**, *cd36* **(H)**, *Mrc1* **(I)**, and *Ym1* **(J)**. n = 5 separate implantations, and 3 experimental repeats. One-way ANOVA followed by Bonferroni’s multiple comparison test; *p < 0.05, **p < 0.01; ns, not significant.

To directly assess whether TRPV4 mediates stiffness-induced phenotypic changes in macrophages, we used an implant model to induce tissue stiffness, and determined the expression levels of cytokines that are linked to macrophage polarization spectrum (20, 35–41). For this, we subcutaneously implanted collagen-coated polyacrylamide hydrogel implants of different stiffness (1 and 50 kPa) in both WT and TRPV4 KO mice, and harvested tissues at 28 days post-implantation. We selected this stiffness range to mimic stiffness of normal skin (~1 kPa) and fibrotic skin (~8–50 kPa). Using qRT-PCR analysis, we observed stiffness-dependent upregulation of two M1 markers, *Il1β* and *Mcp1* in WT tissues, which was absent in TRPV4 KO tissues (**Figures 2A, B**). The expression of additional M1 markers *iNOS*, *Il6*, and *Tnfα* did not exhibit any dependence on TRPV4 under the stiffness conditions assessed (**Figures 2C–E**). We also did not find any significant upregulation in expression levels of M2 markers *Arg1* or *Il10* by stiffer implants in either WT or TRPV4 KO mice, while *Cd36*, *Mrc1*, and *Ym1* expression was reduced in presence of stiffer implants in TRPV4 KO tissues (**Figures 2F–J**). Since skin tissue is a heterogenous environment with complex interactions of many cells, we reasoned that the differential level of this marker expression could be attributed to both macrophages and non-macrophage cells.

### TRPV4 Plays a Role in Matrix Stiffness-Induced M1 Macrophage Polarization *In Vivo*

**Figure 3:**
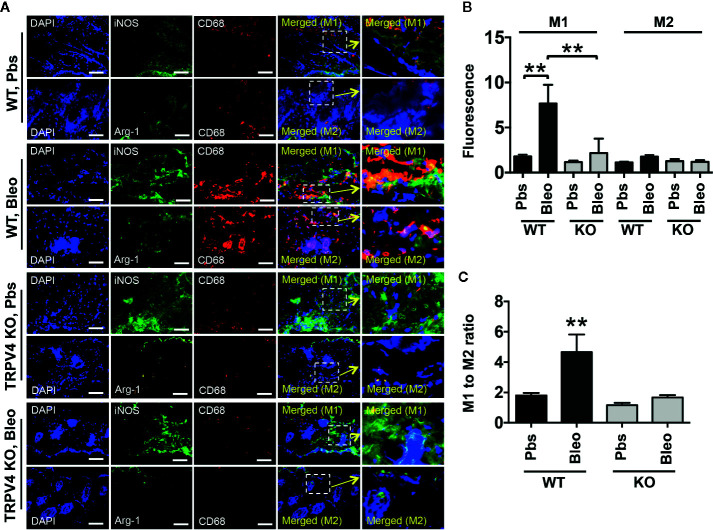
TRPV4 plays a role in matrix stiffness-induced M1 macrophage polarization *in vivo*. **(A)** Representative immunofluorescence images of skin tissues from WT and TRPV4 KO mice subcutaneously injected with bleomycin or saline for 28 days. Seven µm thick tissue sections were co-stained with CD68 (red) plus iNOS (green) (for M1) or CD68 (red) plus Arginase-1 (green) (for M2) as indicated. Marker staining was visualized with Alexa Fluor-conjugated secondary antibody, and nuclei were stained with DAPI. n = 5 mice per group, 5 images were taken for each group. Scale bars: 50 µm. **(B)** Quantification of percentage of co-stained areas in tissues from different treatment groups. **(C)** Quantification of M1/M2 ratios from experiment shown in B. Student’s *t*-test; **p < 0.01.

**Figure 4:**
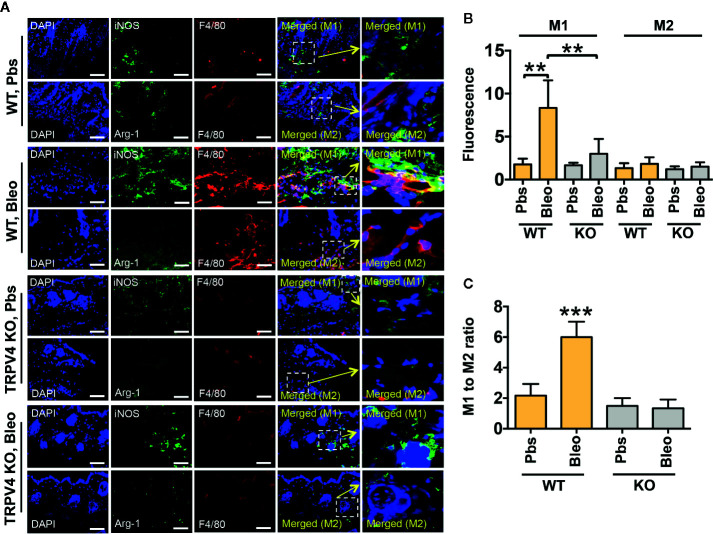
TRPV4 regulates matrix stiffness-induced M1 macrophage polarization *in vivo* as shown by F4/80 staining. **(A)** Skin tissues from WT and TRPV4 KO mice subcutaneously injected with bleomycin or saline for 28 days were co-stained with F4/80 (red) plus iNOS (green) or CD68 (red) plus Arginase-1 (green) as indicated. Marker staining was visualized with Alexa Fluor-conjugated secondary antibody, and nuclei were stained with DAPI. n = 5 mice per group, 5 images were taken for each group. Scale bars: 50 µm. **(B)** Quantification of percentage of co-stained areas in tissues from different treatment groups. **(C)** Quantification of M1/M2 ratios from experiment shown in B. Student’s *t*-test; **p < 0.01, ***p < 0.001.

**Figure 5:**
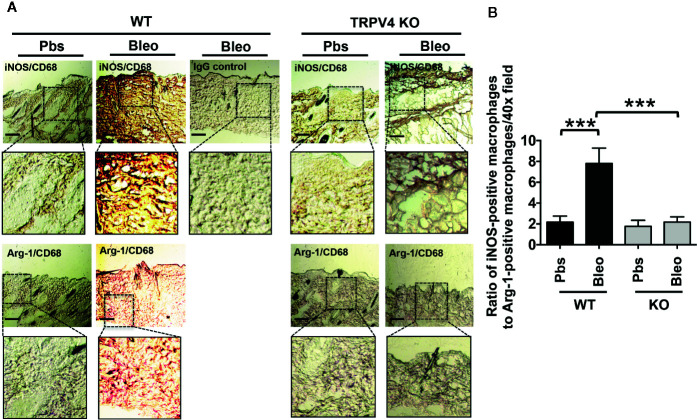
TRPV4 regulates matrix stiffness-induced M1 macrophage polarization *in vivo* as shown by IHC analysis. **(A)** Skin tissues from WT and TRPV4 KO mice subcutaneously injected with bleomycin or saline were co-stained with CD68 (red) plus iNOS (brown) or CD68 (red) plus Arginase-1 (brown) as indicated. IHC with IgGs was used as a negative control. n = 4 images for each group. Magnification: 20×. **(B)** Bar graph shows the ratio of iNOS-positive macrophages to Arg-1-positive macrophages from experiment shown in A. n = 5, Student’s *t*-test; ***p < 0.001.

Polarization of macrophages into M1 or M2 phenotypes is characterized by the differential upregulation of expression of iNOS and Arginase-1, respectively (35–41, 60). However, other cell types also express both antigens. Here, we used a double immunofluorescence staining approach in bleomycin-induced fibrotic skin tissues from WT and TRPV4 KO mice to determine the impact of TRPV4 on the abundance of M1 and M2 macrophages *in vivo*. In our studies, we used co-staining for iNOS or Arginase-1 with CD68 or F4/80 (macrophage markers) as M1 and M2 markers, respectively. We found an increase in the proportion of cells that were double positive for CD68 (or F4/80) and iNOS in the WT tissue treated with bleomycin compared to saline (**Figures 3A–C** and **4A–C**). Although Arginase-1 staining was also prevalent in the WT bleomycin-treated tissue, co-staining with CD68 or F4/80 showed that the upregulation of Arginase-1 was not specific to macrophages. However, loss of TRPV4 function significantly decreased the levels of cells positive for both CD68 (or F4/80) and iNOS in both bleomycin- and saline-treated tissues, with no significant changes in CD68/F4/80/Arg1 positive cells (**Figures 3A–C** and **4A–C**). The M1/M2 ratio in skin indicated a significantly higher M1 macrophage density compared to M2 macrophage density in WT bleomycin-treated tissues, which was completely abrogated by genetic ablation of TRPV4 (**Figures 3C** and **4C**). We further corroborated our immunofluorescence data by IHC analysis of M1 and M2 polarization (**Figure 5**). All together, these data show a possible TRPV4-dependent M1 macrophage polarization spectrum *in vivo* under stiff fibrotic conditions.

### TRPV4 Is Essential for Regulation of Matrix Stiffness-Induced Macrophage M1 Polarization *In Vitro*

**Figure 6:**
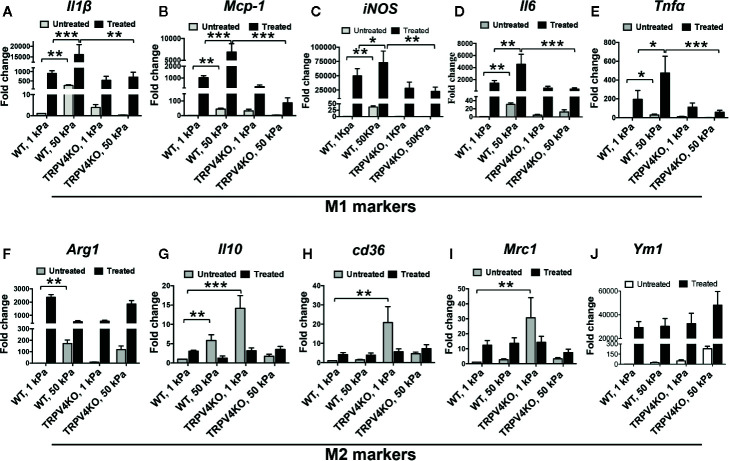
TRPV4 is an essential regulator of matrix stiffness-induced macrophage M1 polarization *in vitro*. Total mRNA was harvested from macrophages seeded on collagen-coated 1 or 50 kPa polyacrylamide hydrogels and treated with LPS (100 ng/ml) plus IFN*γ* (10 ng/ml), or with IL4 (20 ng/ml) plus IL13 (20 ng/ml), or vehicle (untreated). Expression levels (qRT-PCR analysis) of *Il1β* **(A)**, *Mcp-1* **(B)**, *iNOS* **(C)**, *Il6* **(D)**, *TNFα* **(E)**, *Arg1* **(F)**, *Il10* **(G)**, *cd36* **(H)**, *Mrc1* **(I)**, and *Ym1* **(J)**. n = 2 biological repeats and 3 experimental repeats. One-way ANOVA followed by Bonferroni’s test; *p < 0.05, **p < 0.01, ***p < 0.001.

Cells reside in a highly complex tissue microenvironment *in vivo* where they are exposed to an interplay of biophysical and biochemical cues. To mimic the *in vivo* condition, macrophages were plated on two collagen-coated polyacrylamide hydrogels of varying stiffness (1 and 50 kPa) and treated with M1 (IFNγ + LPS) or M2 induction factors (IL4 + IL13) to prime the cells toward an M1 or M2 phenotype (35–37, 41, 60). Using qRT-PCR analysis, we found a significant upregulation in the mRNA expression levels of five well-recognized M1 markers *IL1β*, *Mcp1*, *iNOS*, *Il6*, and *Tnfα* with high stiffness (50 kPa) compared to low stiffness (1 kPa) in WT macrophages (**Figures 6A–E**). Priming the cells with M1-inducing soluble factors increased the levels of expression of M1 markers even further (**Figures 6A–E**). These stiffness and soluble factor-mediated changes in M1 marker expression were diminished with the loss of TRPV4 (**Figures 6A–E**). In contrast, upon induction with high stiffness alone or in combination with M2-inducing soluble factors, the expression level of *Arg1*, *Cd36*, *Mrc1*, and *Ym1* did not show significant upregulation in WT macrophages (**Figures 6F, H–J**). *Il10* levels were increased with high stiffness (**Figure 6G**). Also, deletion of TRPV4 had no significant effect on the levels of expression of these M2 markers (**Figures 6F–J**). Taken together, these data suggest that high matrix stiffness alone or in combination with soluble factors prime macrophages toward an M1 phenotype in a TRPV4-dependent manner.

### TRPV4 Is Directly Involved in Matrix Stiffness-Induced M1 Marker Expression in Macrophages

**Figure 7:**
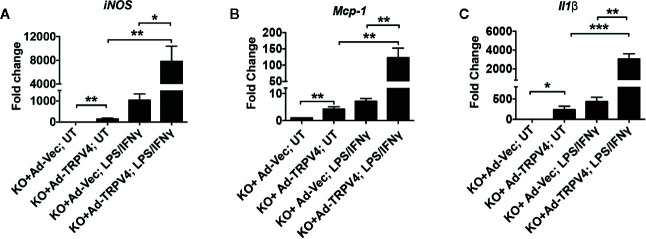
TRPV4 plays a direct role in matrix stiffness-induced M1 marker expression in macrophages. Total mRNA was harvested from TRPV4 KO macrophages infected with Ad-TRPV4 or Ad-Vec control and seeded on collagen-coated 50 kPa polyacrylamide hydrogel and treated with LPS (100 ng/ml) plus IFN*γ* (10 ng/ml), or with vehicle. Expression of *iNOS* **(A)**, *Mcp-1* **(B)**, and *Il1β* **(C)** were analyzed by qRT-PCR and expressed as fold change. n = 2 biological repeats and 3 experimental repeats. One-way ANOVA followed by Bonferroni’s multiple comparison test; *p < 0.05, **p < 0.01, ***p < 0.001.

To determine if the M1 polarization spectrum due to high stiffness was directly related to TRPV4 activity, a gain-of-function approach was used. For this, TRPV4 KO macrophages infected with Ad-TRPV4-WT or Ad-Vec control constructs were plated on high stiffness (50 kPa) collagen-coated polyacrylamide hydrogels either in the presence or absence of M1-inducing factors (LPS + IFNy). The mRNA expression levels of three M1 markers (*IL1β*, *Mcp1*, and *iNOS*) were analyzed by qRT-PCR. In the cells without the M1-inducing factor treatment, TRPV4 overexpression alone increased the expression levels of all three markers significantly compared to Ad-Vec control (**Figures 7A–C**). In M1-inducing factor-treated cells, there was a basal level of expression of all three markers in the Ad-Vec control infected cells because of the treatment. This response was further augmented significantly in cells overexpressing TRPV4 (**Figures 7A–C**). Taken together, these data suggest a direct role of TRPV4 in stiffness and soluble factor-induced M1 macrophage polarization spectrum.

## Discussion

The major findings of the current study are: (a) fibrosis-induced skin tissue stiffening is dependent on TRPV4 as determined by atomic force microscopy; (b) stiffness-dependent switch of macrophages to the M1 phenotype is reliant on TRPV4 *in vivo*; (c) soluble factors and increased substrate stiffness can act in a synergistic manner to upregulate the expression of macrophage M1 markers *in vitro* in a TRPV4-dependent manner; and (d) absolute TRPV4 dependence of matrix stiffness-induced expression of M1 markers was shown by gain-of-function of TRPV4 in TRPV4 KO macrophages.

During inflammatory conditions the extracellular matrix undergoes remodeling that can change its inherent stiffness. Such changes in turn can modulate the function of tissues and tissue-adherent cells in pathological conditions like tumor development, fibrosis, atherosclerosis, and foreign body response (1–4, 27–29). Emerging data suggest that the changes in the extracellular matrix can impact the phenotype and functions of immune cells like macrophages, thereby deregulating the process of inflammation (31–35). Precise understanding of the relationship between abnormal tissue stiffness and inflammation during pathological conditions can be useful in the fields of tissue engineering, fibrosis, tumor development, wound healing, and for the development of targeted therapeutics. Given the mechanosensitive role of TRPV4 and its ubiquitous expression, it is not surprising that TRPV4 has been implicated in disease processes such as fibrosis, foreign body response, and cancer, all of which are associated with changes in tissue stiffness (20, 30, 48, 57, 61). Our previous findings show that genetic ablation of TRPV4 is linked to reduced deposition of collagen and decreased skin fibrosis in a profibrotic drug (bleomycin)-induced mouse model (57). Using atomic force microscopy, we have now extended these findings. Here, we directly measured skin tissue stiffness, and found a wider range of stiffness heterogeneously distributed in and around the fibrotic areas in WT mice compared to TRPV4 KO mice. We used this skin fibrosis model in which skin tissue becomes progressively stiffer over time (62) to test the hypothesis that TRPV4 plays a role in matrix stiffness-induced macrophage polarization spectrum.

Emerging data suggest a role of mechanical factors including matrix stiffness in regulation of cellular phenotype and functionality (1–12). Given their broad tissue distribution profile, macrophages are subjected to wide variations in tissue stiffness ranging from 17 Pa in fat, 1 kPa in skin, 310 mPa in tendons under normal physiological conditions, to 8–50 kPa in fibrotic skin or lung tissues (1–4, 27, 42, 63). Thus, for both our *in vitro* and implant-induced *in vivo* studies, we chose a stiffness range that encompasses both physiological (1 kPa) and pathological (50 kPa) stiffness. Using qRT-PCR analysis, we showed that stiffer implants (50 kPa) upregulated expression of two M1 markers, *Il1β* and *Mcp1*, in implant-adherent skin tissues in a TRPV4-dependent manner. The expression of additional M1 markers, *iNOS*, *Il6*, and *Tnfα*, was independent of TRPV4 under the stiffer condition. Further analysis showed no significant implant stiffness-dependent upregulation of expression levels of M2 markers, *Arg1* or *Il10*, in either WT or TRPV4 KO tissue. However, expression levels of *Cd36*, *Mrc1*, and *Ym1* were reduced under stiffer conditions in TRPV4 KO tissues. These results suggest that TRPV4 possibly impacted the expression of both M1 and M2 markers in response to matrix stiffness *in vivo*. Since skin tissue is a heterogenous organ, and the expression of M1 and M2 markers *in vivo* can be dependent on interactions of many cells, we reasoned that the differential expression of M1 and M2 markers could be attributed to both macrophage and non-macrophage cells. To resolve this issue, we used double immunofluorescence staining to specifically determine the expression level of iNOS (M1 marker) and Arginase-1 (M2 marker) in CD68^+^F4/80^+^-macrophages in fibrotic (stiff) and normal (soft) skin tissues. Our results showed an increase in the proportion of M1 macrophages as evidenced by the abundance of CD68^+^F4/80^+^- plus iNOS double positive cells in stiff *vs.* soft WT tissue. However, a deficiency of TRPV4 function significantly decreased the number of CD68^+^F4/80^+^-/iNOS positive cells in both stiff and soft conditions, but no significant changes in CD68^+^F4/80^+^-/Arg1 positive cells. All together, these results and data from our IHC analysis suggest that stiff matrix promotes M1 macrophage polarization spectrum in a TRPV4-dependent manner.

We performed more controlled *in vitro* experiments where macrophages were exposed to both soluble factors and pathophysiologically relevant substrate stiffness separately, and in combination, to determine the impact of the combination on M1 and M2 marker expression. Our results showed that stiff stiffness (50 kPa) alone increase expression of M1 markers *Il1β*, *Mcp1*, *Il6*, *iNOS*, and *Tnfα* compared to soft stiffness (1 kPa) in WT macrophages, and this response was further augmented by the addition of soluble factors. Intriguingly, we found that deletion of TRPV4 in macrophages significantly decreased both stiffness- and soluble factor-induced upregulation of expression of M1 markers, suggesting a critical role of TRPV4 in this process. In contrast, stimulation of macrophages by soluble factors alone or in combination with high stiffness did not change the expression levels of M2 markers (*Arg1*, *Cd36*, *Mrc1*, and *Ym1*) in a TRPV4-dependent manner. These data suggest that high matrix stiffness alone or in combination with soluble factors prime macrophages toward an M1 phenotype in a TRPV4-dependent manner. An absolute requirement for TRPV4 in M1 macrophage polarization spectrum in response to high stiffness was evident from the results of gain-of-function assays where reintroduction of TRPV4 significantly augmented the expression levels of M1 markers *iNOS*, *Mcp1*, and *Il1β* in TRPV4 KO macrophages.

Although substrate stiffness has been shown to regulate macrophage functions including polarization, there remains a paucity of data exploring the underlying molecular mechanisms involved in mechanical cue-induced phenotypic and functional change in macrophages (31–35). One report has shown that macrophages grown on stiff (323 kPa) polyacrylamide hydrogels predominantly adopted a M1 phenotype with decreased phagocytic ability and migration capacity compared to macrophages grown on softer (11 and 88 kPa) gels (34). Others have reported a role of cytoskeletal modifications and small Rho GTPase activity in shape- and substrate-induced macrophage polarization (19). In contrast, a recent report showed that increased substrate stiffness achieved through a modification of collagen matrices shifted macrophages toward an M2 or anti-inflammatory phenotype with an increase in IL10/IL12 cytokine secretion profile (43). There has been a growing interest in understanding the role of mechanosensitive TRP proteins in various macrophage functions. It has been shown that IFN*γ*-mediated M1 priming of macrophages was dependent on TRPC1-mediated calcium influx (64). TRPM7 was also implicated in both the polarization spectrum and proliferation of M2 macrophages (46). Previously it was shown that increased stiffness of the PEG-RGD substrate alone had no significant effect on macrophage responsiveness, but the concerted action of stiffness and soluble stimulants induced the M1 phenotype and increased the levels of TNFα, IL1*β*, and IL6 (5). Discrepancies between this prior study and our current study could be a consequence of differences in the range of stiffnesses and the substrates used in the studies. Contrary to our current findings, TRPV4 was found to attenuate oxidative stress-mediated M1 macrophage polarization spectrum in an experimental model of non-alcoholic steatohepatitis liver (65). Such differential regulation of macrophage polarization spectrum by TRPV4 could be due to differences in the pathways in liver tissue that mediate responses to oxidative stress. Further, we cannot exclude the possibility that the phenotypic shift in macrophages in global TRPV4 knockout mice may be related to developmental compensation or unknown secondary compensation. However, previous studies from our laboratory showed that the absence of TRPV4 did not impair basal macrophage maturation *in vivo* (20). We used CD68 and F4/80 to identify tissue macrophages, two widely used markers for identifying macrophages in both *in vivo* and *in vitro* setting. Since macrophages exist in an array of activation states, it is possible that *in vivo* CD68^+^F4/80^+^-macrophages may represent specific and special subpopulations of macrophages. Moreover, despite the known low stiffness of fat tissue, the resident macrophages present in chronic, non-resolving diabetic wounds or inflamed adipose tissue express mainly “M1” markers (66, 67). However, macrophages present in normal healthy adipose tissue express mainly “M2” markers (67). Thus, one can speculate that under certain pathological conditions including diabetic wounds or inflamed adipose tissue, a concerted action of soluble factors and matrix stiffness can play a role in determining the macrophage phenotype. Future research would shed light on this area by unveiling some exciting pieces of the puzzle.

In summary, the results of our study provide compelling evidence that stiff matrix alone or in concert with soluble factors leads to M1 macrophage polarization spectrum in a TRPV4-dependent manner. Thus, our study provides insights into the matrix stiffness-macrophage interaction, which may be utilized in the development of bio-competent implants and therapeutics.

## Data Availability Statement

The original contributions presented in the study are included in the article/supplementary material. Further inquiries can be directed to the corresponding author.

## Ethics Statement

All animal experiments were performed following University of Maryland review committee-approved protocols in accordance with guidelines of the Institutional Animal Care and Use Committee (IACUC).

## Author Contributions

SR, RG, and BD conceived the study, designed and performed the experiments, and analyzed data. SR and BD wrote the manuscript. All authors contributed to the article and approved the submitted version.

## Funding

This work was supported by NIH (R01EB024556) and NSF (CMMI-1662776) grants to SR.

## Conflict of Interest

The authors declare that the research was conducted in the absence of any commercial or financial relationships that could be construed as a potential conflict of interest.
